# The history of cryptosporidiosis

**DOI:** 10.1186/s13071-026-07498-8

**Published:** 2026-07-18

**Authors:** Dietmar Steverding

**Affiliations:** Bob Champion Research and Education Building, Norwich Medical School, University of East Anglia, Norwich Research Park, Rosalind Franklin Road, Norwich, NR4 7UQ UK

**Keywords:** Cryptosporidiosis, *Cryptosporidium* spp., *C. parvum*, *C. hominis*, Outbreaks

## Abstract

In this review, the history of cryptosporidiosis caused by parasites of the genus *Cryptosporidium* is outlined. These apicomplexan parasites evolved together with their vertebrate host during the early Cambrian period. The main etiological agents of human and bovine cryptosporidiosis are the zoonotic species *C. parvum* and the anthroponotic species *C. hominis*. Palaeoparasitological data indicate that humans have been parasitised by cryptosporidia since ancient times. Although the first *Cryptosporidium* species was described in 1907, the protozoan was only recognised as a gastrointestinal parasite of humans and cattle in the 1980s. The parasite became known to the general public primarily through media reports of waterborne outbreaks of gastroenteritis. Cryptosporidiosis is now acknowledged as a leading cause of diarrhoeal disease worldwide, with severe health implications for young, malnourished children and immunocompromised patients, and with severe economic consequences for cattle farmers.

**Graphical Abstract:**

**Figure:**
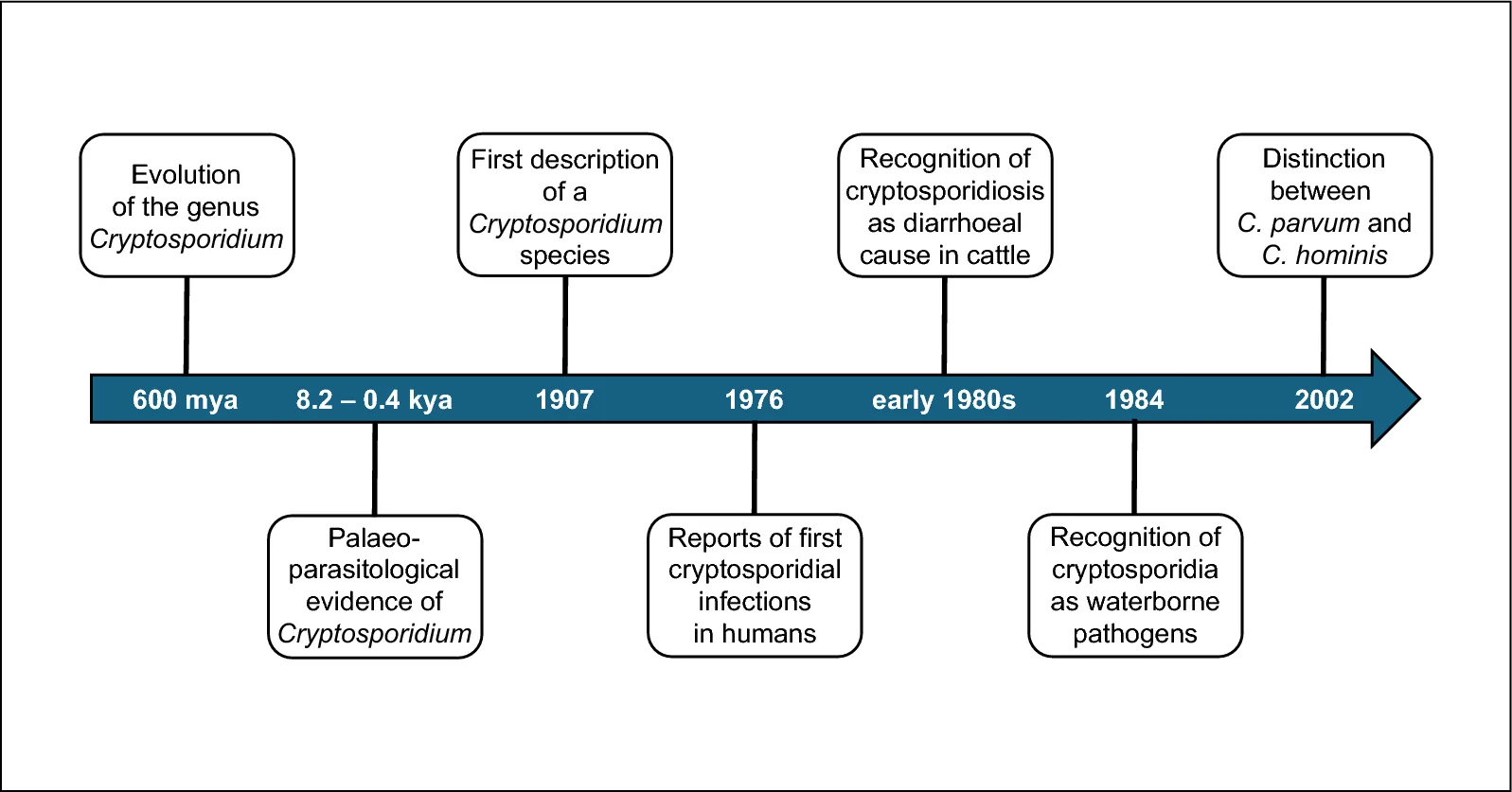

## Background

The aetiological agents of cryptosporidiosis are protozoan parasites of the genus *Cryptosporidium*. The infectious disease can cause respiratory and gastrointestinal illness in humans and animals. It has been estimated that cryptosporidiosis is responsible for more than 8 million cases of diarrhoea in humans worldwide each year [1]. In particular, cryptosporidiosis is a major cause of diarrhoea-related morbidity and mortality in children under 5 years of age [2]. In addition, the disease is also a common cause of diarrhoea in livestock, especially in dairy calves [3]. Livestock cryptosporidiosis not only has a significant economic impact on farmers but can also act as a zoonotic reservoir for infecting humans.

There are at least 47 different *Cryptosporidium* species and more than 120 distinct genotypes, of which 19 species and 4 genotypes have been found to infect humans [4, 5]. *C. hominis* and *C. parvum* are responsible for the majority of human cryptosporidiosis cases, while *C. meleagridis, C. felis, C. canis*, and *C. ubiquitum* are the next four most common species causing the disease [4]. The four main *Cryptosporidium* species infecting cattle are *C. parvum*, *C. bovis*, *C. ryanae*, and *C. andersoni* [5]. Whereas *C. parvum* is most prevalent in high-income countries, especially in pre-weaned calves, *C. bovis* is the dominant species in nursing calves in low-income countries [6]. Generally, *C. bovis*, *C. ryanae*, and *C. andersoni* are more commonly found in older calves and adult cattle [7].

Humans and animals become infected with cryptosporidia when they ingest fully sporulated oocysts in food or drinking water contaminated with faeces from infected hosts. Cryptosporidia oocysts can survive for several months in water and moist environments and are resistant to strong disinfectants such as chlorine [8]. Once the oocysts reach the upper small intestine, they excyst and release 4 sporozoites, which attach themselves to enterocytes. The parasitised epithelial cells form a parasitophorous vacuole around the protozoan in which the parasite differentiates into a meront. Within the parasitophorous vacuole, the meront undergoes asexual multiplication and produces 8 merozoites After the merozoites have escaped the parasitophorous vacuole, they can infect nearby enterocytes to complete another round of asexual multiplication. Alternatively, meronts can develop into male and female gamonts. Male gametes released by male gamonts fertilise female gametes, which then form zygotes. Subsequently, the diploid zygote develops into an oocyst containing 4 sporozoites. Oocysts can either excyst internally to autoinfect the host or be excreted with the faeces into the environment to infect new hosts [9]. (A more detailed description of the reproduction of cryptosporidia is given in Sect.  4 in connection with the discovery of the life cycle.)

The clinical presentation of infections with cryptosporidia varies depending on the immune status of the patient [10]. In immunocompetent individuals, the disease may present with a bout of watery diarrhoea. In most cases, human cryptosporidiosis is self-limiting and resolves within 2–3 weeks. Individuals with a weakened immune system (e.g., HIV/AIDS, cancer, and transplant patients) may experience severe complications, including life-threatening malabsorption and wasting. The clinical symptoms of bovine cryptosporidiosis caused by *C. parvum* are watery diarrhoea, abdominal pain, depression, and anorexia [11]. Calves infected with *C. parvum* are usually dehydrated, anorexic, and lethargic [11]. Watery diarrhoea in calves can be mild to severe and is pale-yellow with mucus [11]. The medical manifestations of infections with C. *andersoni* in cattle are mainly moderate to severe reduced weight gain and feed inefficiency, resulting in decreased milk production in dairy cows [11]. *C. bovis* and *C. ryanae*, however, often cause only subclinical infections in weaning calves and older cattle [12]. In addition, there is evidence that infections with cryptosporidia can lead to dysbiosis with reduced bacterial diversity and that probiotics can alleviate the pathological impact of the pathogen [13].

### Evolution of *Cryptosporidium* parasites

Cryptosporidia belong to the phylum Apicomplexa, which evolved about 1 billion years ago from free-living photosynthetic algae [14]. A more recent study suggests that the parasitism of cryptosporidia coevolved with the origin of the vertebrates around 600 million years ago [15]. It was also found that when the crown mammalian and avian orders started to develop at the end of the Cretaceous period, cryptosporidia also began to diversify [15]. It therefore seems that the rapid increase in new mammalian and avian hosts sparked the adaptive radiation of the genus *Cryptosporidium*. In addition, a significant correlation between cryptosporidia and vertebrate hosts’ phylogenetic trees was observed, supporting a common macroevolutionary scenario amongst them [15].

**Fig. 1:**
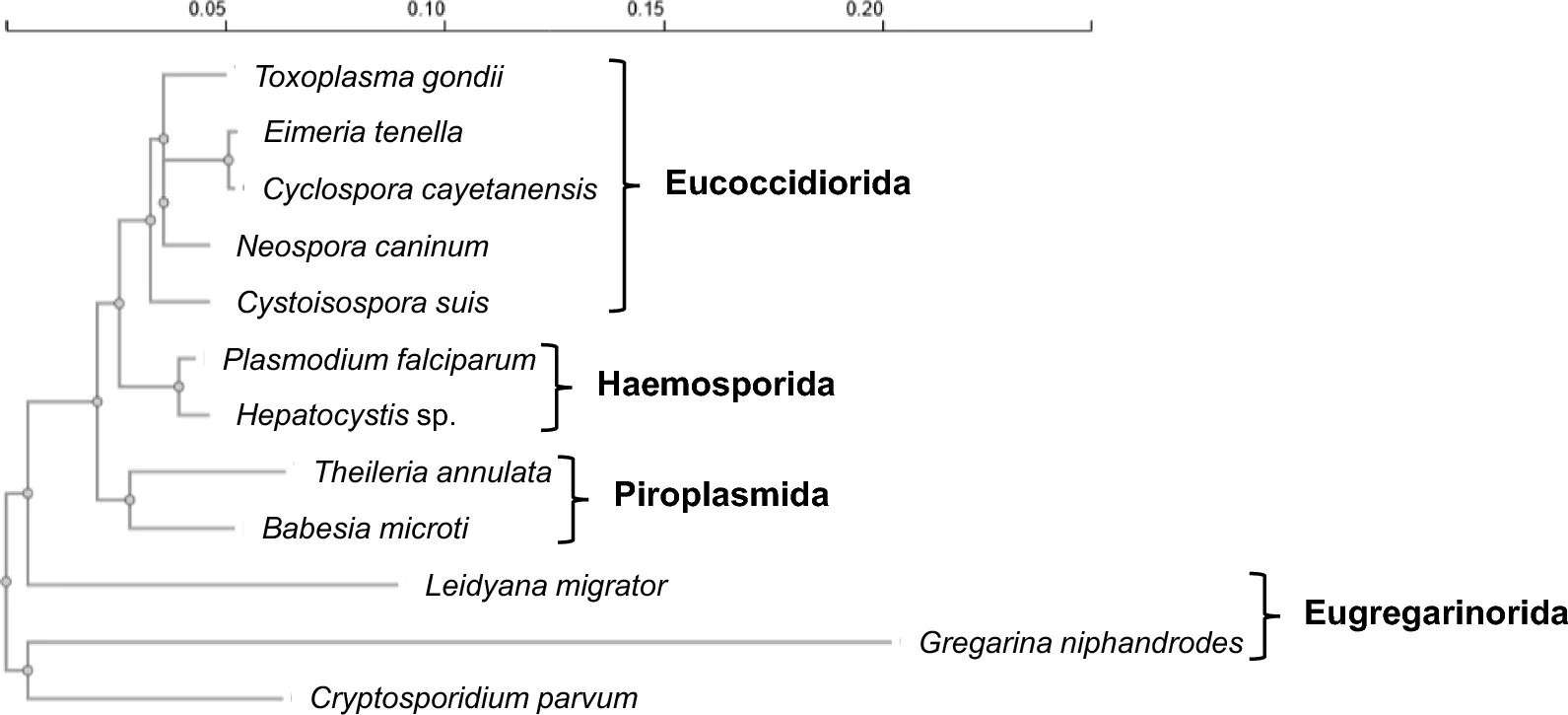
β-tubulin complete protein sequence phylogenetic tree of apicomplexan parasites. The phylogenetic dendrogram was calculated using the neighbour-joining method with the programme Clustal Omega [25]. Orders to which the apicomplexan organisms belong are indicated. The final version of the figure was prepare using PowerPoint

Originally, the genus *Cryptosporidium* was placed in the coccidian clade based on life cycle features and the development of sporozoites either within environmentally resistant oocysts or sporocysts [16]. However, the phylogenetic position of the genus *Cryptosporidium* within the coccidian clade has been questioned. Firstly, phylogenetic analyses have shown that the subclass of Coccidia (Coccidiasina) is only monophyletic if the genus *Cryptosporidium* is excluded [17–19]. Instead, the genus *Cryptosporidium* seems to be a sister group of Coccidia and Haematozoa [14, 18]. Secondly, subsequent phylogenetic analyses suggest that the genus *Cryptosporidium* is more closely related to the species of the subclass Gregarinia (Gregarinasina) [20–23]. Furthermore, phylogenetic analyses using fused small and large ribosomal RNA subunit sequences and six protein sequences (α- and β-tubulin, cyclin-dependent protein kinase (cdc2), dihydrofolate reductase-thymidylate synthase (DHFR-TS), cytosolic 70 kDa heat-shock protein (HSP70), and elongation factor EF-1α) indicated an early emergence of the genus *Cryptosporidium* at the base of the Apicomplexa phylogenetic tree [24]. The re-evaluation of the phylogenetic relationship of apicomplexan β-tubulin sequences, including two eugregarine sequences, with the Clustal Omega multiple sequence alignment programme [25], corroborates an early branching of the genus *Cryptosporidium* and a close relatedness with the genus *Gregarina* (Fig. 1). Moreover, it appears that the genus *Cryptosporidium* could be used as an outgroup to determine the phylogenetic positions of all other apicomplexan parasites. To establish the true position of the genus *Cryptosporidium* within the apicomplexan tree, further phylogenetic analyses of conserved protein and gene sequences are required.

The particular position of the genus *Cryptosporidium* within the Apicomplexa could also be due to the fact that the species of this protozoan group do not possess mitochondria and apicoplasts. While most of the extant apicomplexan species have a single canonical mitochondrion, although with drastically reduced genomes encoding just 3 proteins (cytochrome c oxidase subunits 1 and 3 and cytochrome b) and several highly fragmented ribosomal RNAs, cryptosporidia possess only mitochondria-related organelles (MROs) that lack mitochondrial DNA [26, 27]. Even within the *Cryptosporidium* genus, MROs are quite variable. For example, the intestinal infecting species *C. parvum* and *C. hominis* have greatly reduced MROs (mitosomes) that function only in the biosynthesis of Fe-S clusters, while the stomach infecting species *C. muris* and *C. andersoni* harbour MROs with a functional Krebs cycle and an incomplete electron transport chain (complexes I, II, and V) [27, 28]. There is evidence that the MROs of cryptosporidia are secondarily reduced relict mitochondria. For instance, the mitochondrial origin of the mitosome in *C. parvum* was confirmed by the presence of the mitochondrial marker proteins chaperonin 60 (Cpn60) and chaperone HSP70 (mtHSP70) in the relict organelle [29, 30]. In the case of mtHSP60, it was also shown that its gene is of proto-mitochondrial origin [30]. The absence of plastid-like DNA and any physical structure representing a plastid organelle in cryptosporidia led to the conclusion that species of this apicomplexan genus do not harbour a relict apicoplast [31–34]. However, 7 genes have been identified in *C. parvum* to be likely of plastid/endosymbiont origin [34]. Taken together, these findings suggest that *Cryptosporidium* species evolved from a mitochondria- and plastid-containing ancestor and lost these organelles during their adaptation to their vertebrate hosts.

**Fig. 2:**
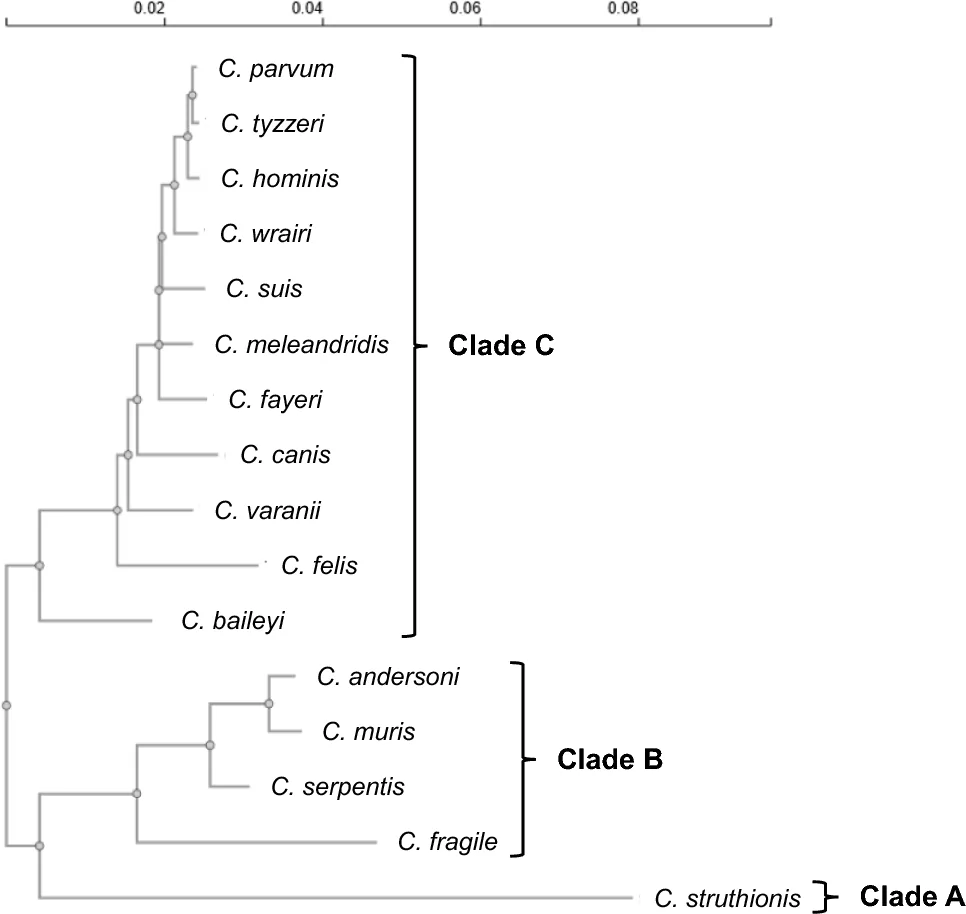
Phylogenetic tree of *Cryptosporidium* species based on complete 18S rRNA sequences. The phylogenetic dendrogram was calculated using the neighbour-joining method with the programme Clustal Omega [25]. The final version of the figure was prepare using PowerPoint

The phylogenetic analysis of complete 18S rRNA sequences using the Clustal Omega programme confirmed previous findings obtained by sequence alignment of concatenated gene fragments of 18S rRNA, actin, and hsp70 that *Cryptosporidium* species group into three clades [15] (Fig. 2). The first clade, A, consists only of *C. struthionis*; the second clade, B, of *C. fragile*, *C. serpentis*, *C. andersoni*, and *C. muris*; while the third clade, C, includes all the other species (Fig. 2) [15]. Whereas in the analysis using the Clustal Omega programme *C. parvum* appears to be most closely related to *C. tyzzeri* (Fig. 2), in a previous study the nearest relatives of *C. parvum* were found to be *C. hominis* and *C. cuniculus* [15]. As the split between *C. hominis* and *C. cuniculus* was determined to have occurred around 6 million years ago, the time when the Hominini lineage diverged into the *Homo* genus and the *Pan* genus (chimpanzees and bonobos) [35], it can be assumed that this *Cryptosporidium* species had adopted human ancestors as hosts and was tracing human evolution [15]. A recent genomic study found that *C. hominis* had diverged into two subspecies, *C. h. aquapotentis* and *C. h. hominis*, about 500 years ago [36]. The two subspecies exhibit distinct biological characteristics and demographic patterns [36]. A similar event of a recent divergence into two subspecies with distinct host associations was also reported for *C. parvum* [37]. While one subspecies, *C. p. parvum*, is responsible for zoonotic transmission, the other subspecies, *C. p. anthroponosum*, is mainly spread between humans [37]. Although the evolutionary mechanisms that lead to the emergence of the *C. hominis* and *C. parvum* subspecies are unclear, genetic recombination at species and subspecies levels may have driven the development of *Cryptosporidium* subspecies with greater human specificity, virulence, and transmissibility [36, 37].

### Historical evidence of *Cryptosporidium* parasites

#### Prehistory (before 3000 BCE)

**Fig. 3:**
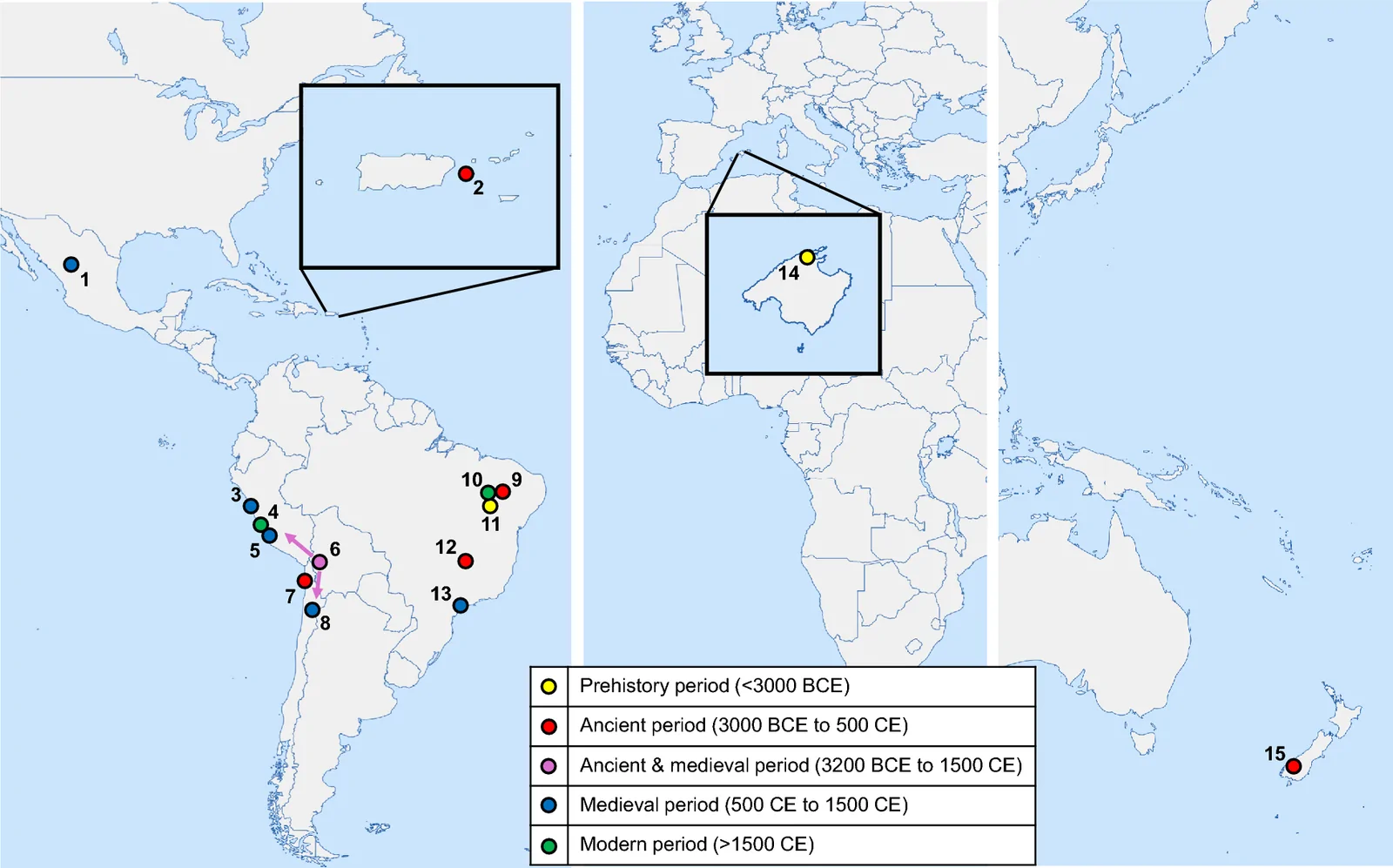
Locations of historical evidence of *Cryptosporidium* spp. in the world. 1, La Cueva de los Muertos Chiquitos (Durango); 2, Sorcé (Vieques); 3, PV35-4 (Huarmey Valley); 4, Vale do Rio Chillon (Lima); 5, Huayuri (Ica Region); 6, South American Andes; 7, Caserones-Tarapacá 40 (Iquique); 8, San Pedro de Atacama (Antofagasta Region); 9, Boqueirão da Pedra Furada (Serra da Capivara National Park); 10, Toca da Extreme II (Serra da Capivara National Park); 11, Toca da Baixa dos Caboclos (Serra da Capivara National Park); 12, Gruta do Gentio II (Unaí); 13, Sítio Fonseca (São Paulo); 14, Cova Estreta (Pollença, Mallorca); 15, Dart River Valley (Glenorchy, South Island). The map templates are from MapCharts (https://www.mapchart.net), which are freely available under a Creative Commons Attribution-ShareAlike 4.0 International License (CC BY-SA 4.0). The final version of the figure was prepare using PowerPoint

The earliest palaeoparasitological evidence of cryptosporidia comes from the Boqueirão da Pedra Furada archaeological site located in Serra da Capivara National Park, in the state of Piauí, Brazil (Fig. 3). This site is known for rock paintings and traces of prehistoric human occupation dating back to 12000 years. At this excavation site, 3 coprolite samples (1 human coprolite and 2 rodent coprolites) between 8170 and 7230 years old (6220–5280 BCE) were found positive for cryptosporidia surface antigen by enzyme-linked immunosorbent assay (ELISA) [38]. Another prehistoric record of cryptosporidia was found on the Mediterranean island of Mallorca. Coprolites of the extinct dwarf goat *Myotragus balearicus*, dating back to 3000 BCE, were recovered from the Cova Estreta located on the northern coast of the island (Fig. 3). Of the 25 coprolites analysed, 9 gave a positive result for cryptosporidia antigen by ELISA [39].

#### Ancient history (3000 BCE to 500 CE)

There are a few documented confirmations of cryptosporidia in ancient specimens (Fig. 3). The oldest evidence comes from the Toca da Extreme II archaeological site, also located in Serra da Capivara National Park, Piauí, Brazil. A rodent coprolite dating from 2780 BCE was found positive for cryptosporidia antigen by ELISA [38]. A second description of cryptosporidia from ancient times is from a pottery-making farmer occupation site, Gruta do Gentio II, in the state of Minas Gerais, dating back 3490 years (1540 BCE) [38]. Out of 10 human coprolites, one was positive for the presence of cryptosporidia. Another account describes the identification of cryptosporidia oocysts in faeces of 500- to 3000-year-old Andean mummies (1050 BCE to 1500 CE) [40]. Of the 39 mummies investigated, 15 gave a positive result by immunofluorescence assay (IFA). However, since there was no age information given for the samples, it is not clear which of the positively tested specimens dates back to ancient times. Using DNA analysis, a sequence obtained from a coprolite (out of 4) of the extinct upland moa *Megalapteryx didinus* dating back to the late Holocene age (< 1050 BCE) found in the Dart River Valley of New Zealand’s South Island was identified as belonging to *C. struthionis*, a *Cryptosporidium* species known to infect modern-day ostrich [41]. A fifth identification of cryptosporidia in human coprolites comes from an associated cemetery of a village settlement of the archaeological site of Caserones-Tarapacá 40 in northern Chile from the Formative Period (1100 BCE to 660 CE) [38]. One out of 4 specimens gave a positive result by ELISA testing for the presence of cryptosporidia antigen. One more find of cryptosporidia in an ancient human coprolite originated from the excavation site at the Sorcé settlement in the Caribbean Island of Vieques, Puerto Rico [42]. In 1 out of 10 coprolites radiocarbon dated to 215–475 CE, cryptosporidia DNA was detected by metagenomic sequencing.

#### Middle ages (500 CE to 1500 CE)

There is evidence of the occurrence of cryptosporidia before pre-colonial times in Latin America (Fig. 3). The northernmost detection of the protozoan was made at the archaeological site of La Cueva de los Muertos Chiquitos, Rio Zape Valley, Mexico [43]. The cave was utilised by the Loma San Gabriel people from 600 to 800 CE Overall, 90 coprolites were analysed by ELISA for the presence of cryptosporidia antigen, of which 66 tested positive or likely positive for *C. parvum*. Two further pieces of evidence of cryptosporidia in medieval samples come from two archaeological sites in Peru: the PV35-4 site (Middle Horizon Period, 770–830 CE) located in the desert on the north-central coast of the Huarmey Valley [43] and the Lost City of Huayuri (Late Intermediate Period, 1200–1400 CE) located in the Peruvian coastal desert in the Ica Region [38]. At the first site, 1 out of 22 human coprolites tested positive for cryptosporidia oocysts by IFA [44]. At the second site, cryptosporidia antigen was detected in 2 out of 3 human coprolite specimens [38]. Further human coprolite specimens positive for cryptosporidia antigen and dating back to 900–1450 CE were found at excavation sites of the Chilean town of San Pedro de Atacama [38]. In total, 2 out of 4 samples gave a positive result. Since the age range of the Andean mummies extends back to the Middle Ages (see chapter above) [40], it is likely that some of the cryptosporidia oocysts found in the faeces of mummified bodies are from pre-Columbian times. Another description of cryptosporidia in medieval samples originates from the archaeological site of Sítio Fonseca, São Paulo, Brazil [38]. Four out of 4 human sediment samples dating from 940 CE tested positive for the presence of cryptosporidia antigen by ELISA.

#### Modern times (1500 CE to present)

Evidence of cryptosporidia in specimens younger than 1500 CE comes from two archaeological sites in South America (Fig. 3) [38]. One of the sites is located in the Vale do Rio Chillon (Chillón River Valley) in Peru and dates from the Late Inca Period (1476–1534 CE). One human sediment sample (out of 2) proved positive for cryptosporidia antigen. The other one is the Toca da Baixa dos Caboclos archaeological site located in the Serra da Capivara National Park, Piauí (Brazil), and is 400 years old (1550 CE). Only 1 human coprolite was discovered, which showed a positive result for the presence of cryptosporidia antigen.

**Fig. 4:**
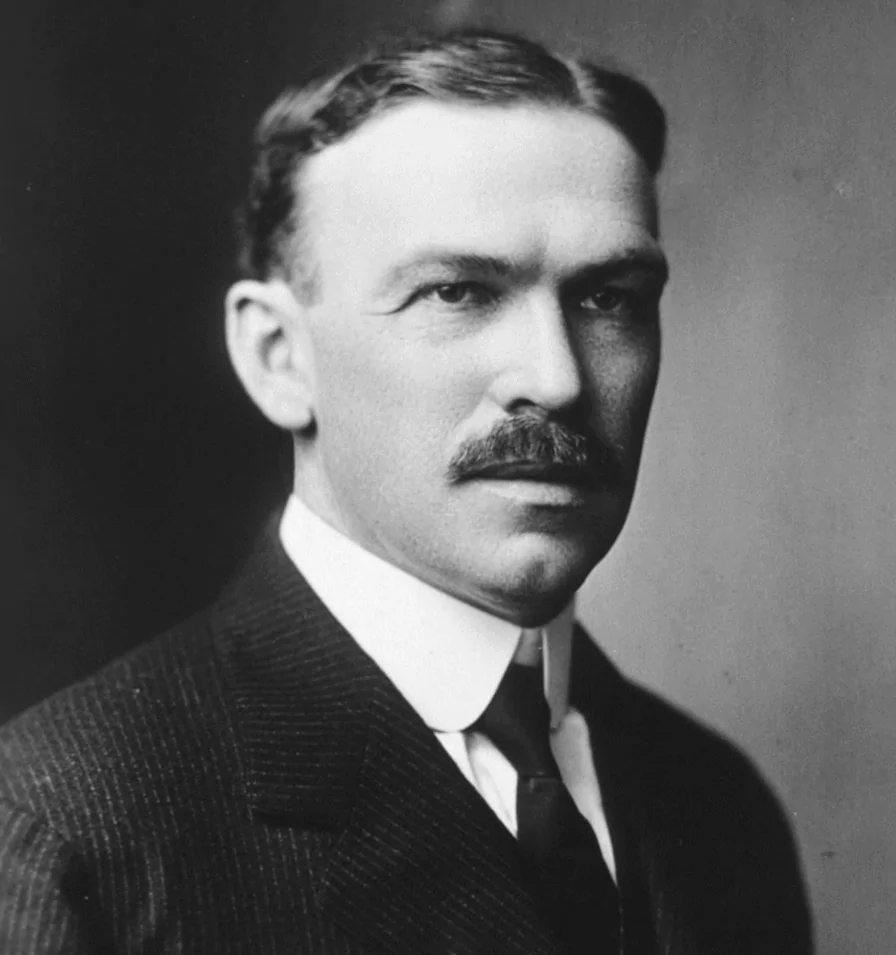
Portrait of Ernest Edward Tyzzer, who discovered *Cryptosporidium muris*. Wikimedia Commons, link: https://commons.wikimedia.org/wiki/File:Ernest_E._Tyzzer.jpg (source: http://resource.nlm.nih.gov/101430621). This file is freely available under the Creative Commons CC0 1.0 Universal Public Domain Dedication

The first description of a *Cryptosporidium* species was made by the American physician, pathologist, and parasitologist Ernest Edward Tyzzer (1875–1965) (Fig. 4) in 1907 when he reported the identification of a new sporozoan found in the peptic glands of a domesticated house mouse [45]. Since he could not find any description of the newly discovered protozoan, he named it *C. muris* [45]. In a subsequent work published in 1910, Tyzzer described in great detail the biology and life history of this newly discovered protozoan [46]. As this new sporozoan lacks sporocysts within the oocysts, Tyzzer suggested that this species should belong to a new genus, which he named *Cryptosporidium* (meaning hidden spore) with *C. muris* being its type species [46]. In 1912, Tyzzer identified another *Cryptosporidium* species, *C. parvum*, in laboratory mice based on the smaller size of its oocysts and its infection site in the small intestine [47]. A third *Cryptosporidium* species, *C. meleagridis*, was identified in 1955 during a diarrhoea outbreak in a flock of turkey poults [48]. The new pathogenic species was isolated from the intestine of infected birds and was associated with acute clinical disease with severe diarrhoea but a low death rate [48]. In 1966, a report was published on the observation of a *Cryptosporidium* organism found in the small intestine of guinea pigs resembling that of *C. parvum* [49]. Later, the cavine species was named *C. wrairi*, as this organism could not infect mice, chickens, turkeys, and rabbits [50]. Cryptosporidial infection of a calf was reported for the first time in 1971 [51]. One year later, two cases of cryptosporidiosis in Rhesus monkeys were described, in which the sporozoan was found in the epithelium of bile, intrahepatic and pancreatic ducts, gall bladder, and small and large intestines [52]. In the following years, further cryptosporidial infections were reported in various animal species, such as sheep [53], geese [54], pigs [55], snakes [56], and horses [57]. The first cryptosporidial infections in humans were reported in 1976 in a 3-year-old girl [58] and in an immunosuppressed patient [59]. By the early 1980s, cryptosporidiosis was increasingly recognised as a chronic and life-threatening disease in AIDS patients [60]. Around the same time, it also became clear that cryptosporidiosis is a major cause of outbreaks of diarrhoea in dairy cattle, in particular in calves [61, 62]. Cryptosporidial parasites were acknowledged as waterborne pathogens for the first time in 1984 during an outbreak in the suburban community of Braun Station in Texas, probably affecting several hundred people [63]. Initially, it was assumed that *C. parvum* was the primary causative agent of human cryptosporidiosis. By the early 1990s, it was discovered that humans were infected with two genetically different types of *C. parvum* [64–66]. One was found exclusively in humans and termed type H or type I, while the other was the same that also infects cattle and was termed type C or type II [67]. In 2002, it was eventually proposed that *C. parvum* type H/1 be a new anthroponotic species, *C. hominis*, and different from the zoonotic *C. parvum* type C/2 [68]. More recently, it has been suggested to further divide *C. hominis* and *C. parvum* into the subspecies *C. h. aquapotentis* and *C. h. hominis*, and *C. p. parvum* and *C. p. anthroponosum*, respectively (see Sect. 2).

### Elucidation of the life cycle of *Cryptosporidium* parasites

**Fig. 5:**
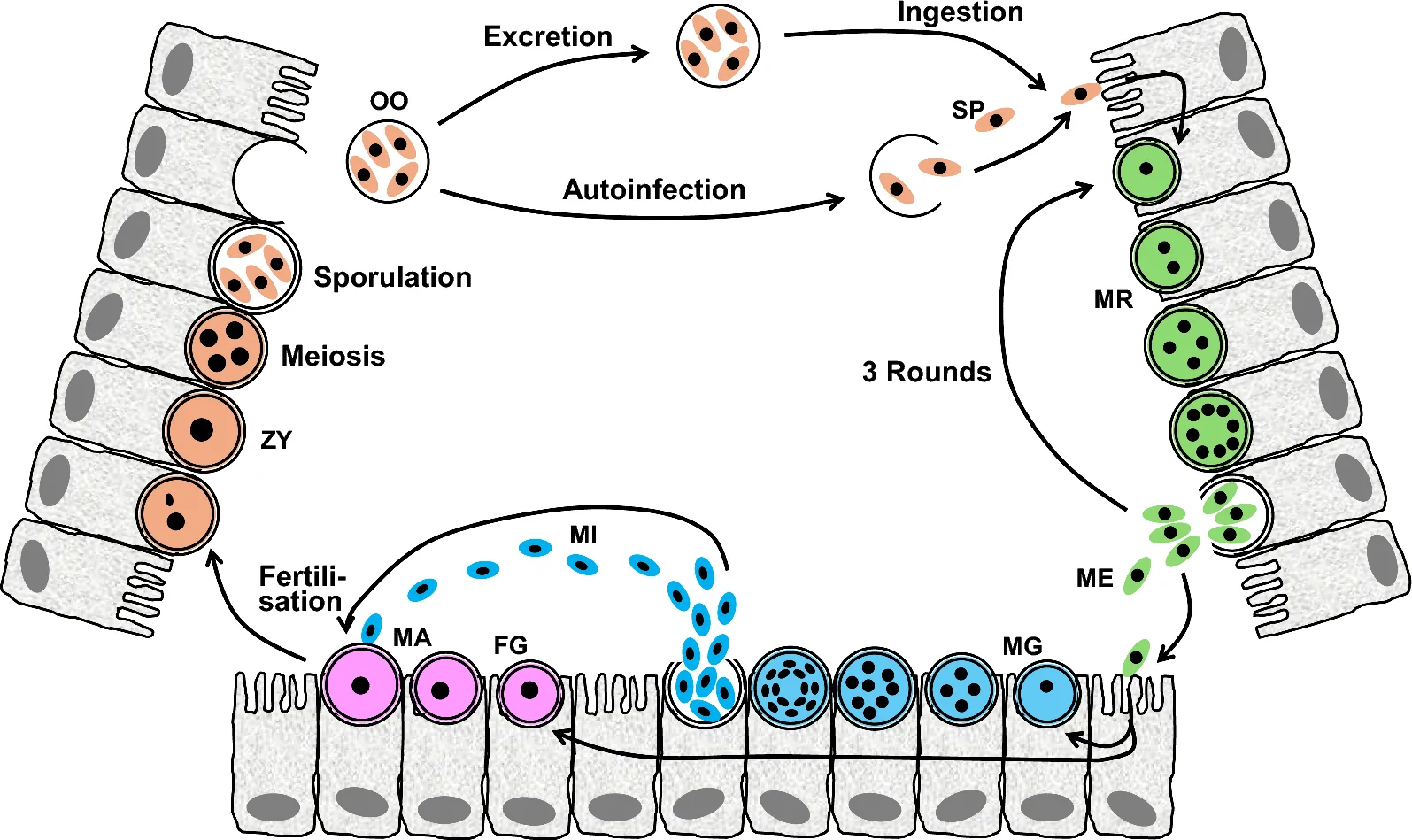
*Cryptosporidium* spp. life cycle. Following ingestion of sporulated oocysts (OO) with faecally contaminated water or food, sporozoites (SP) are released that invade intestinal epithelial cells. The sporozoites develop into meronts (MR) that undergo synchronous merogony (schizogony) to form 8 merozoites (ME). The meront ruptures, and the released merozoites infect neighbouring cells. After 3 rounds of merogony, merozoites undergo gametogony and develop into sexual stages, both male gamonts (MG) and female gamonts (FG). The male gamont undergoes 4 rounds of nuclear division to produce 16 male gametes (microgametes, MI), whereas the female gamont is cell arrested and matures into a female gamete (macrogamete, MA). Released male gametes fertilise intracellular female gametes, and zygotes (ZY) are formed. The zygote undergoes sporogony, and following meiosis and sporulation, an oocyst is produced containing 8 sporozoites. Oocysts are excreted with faeces, resulting in transmission of the parasite to a new host or can autoinfect the same host. Merogony is shown in green, gametogony in blue/red, and sporogony in orange. The figure was created using PowerPoint

In his first reports on *C. muris* and *C. parvum*, Tyzzer already described the fundamental principles of the life cycles of cryptosporidial parasites, which remain generally valid today [45–47]. He identified all life cycle stages: the sexual stages (male and female gamonts producing 16 male gametes and a single female gamete, respectively), the oocyst (containing 4 sporozoites), and the asexual meront (producing 8 merozoites). Tyzzer also recognised that oocysts are the infectious stages and may be responsible for autoinfection. Although Tyzzer thought that the entire development of cryptosporidia was extracellular and that the parasites lived on the surface of intestinal epithelial cells, in 1966 it was shown that *C. parvum* is an intracellular parasite [69]. However, the localisation of the parasites is extracytoplasmic at the apical surface of epithelial cells in a parasitophorous vacuole composed of host and parasite membranes [70, 71]. Since the unique intracellular localisation of cryptosporidia could only be determined by high-resolution electron microscopy, a technique developed in the 1940s, Tyzzer could not ascertain this with the method available to him at the time. In 1971, Vetterling et al. introduced the concept of two merogonic generations: a type I meront with 8 merozoites (the one described by Tyzzer) and a type II meront with 4 merozoites [50]. Type II meronts are formed from type I meronts, but instead of continuing the asexual cycle, they develop into male and female gamonts, respectively. The concept of the two-meront model has become the textbook knowledge for the life cycle of cryptosporidia ever since. More recent research, however, has found no evidence for a type II meront [72]. The type II meronts observed by Vetterling and colleagues were most likely immature oocyst stages [72]. However, live cell imaging revealed that cryptosporidia actually perform 3 asexual replication rounds, directly followed by 1 sexual reproductive cycle without involving a distinct intermediate type II meront stage (Fig. 5) [72]. Another enigma of cryptosporidia that found its way into textbooks is an observation made in 1986 that zygotes can develop into thick-walled or thin-walled oocysts [71]. It was suggested that thick-walled oocysts are secreted and transmit the infection to a new host, whereas thin-walled oocysts are responsible for autoinfection [71]. However, experimental confirmation of morphologically and functionally different oocysts is lacking. The latest findings confirm that Tyzzer was all along correct with his description of the life cycle of cryptosporidia over 100 years ago. This is all the more remarkable considering that the life-cycle stages of this organism are barely visible under the light microscope.

### Outbreaks of human cryptosporidiosis

In 1989 and 1993, two large outbreaks of human cryptosporidiosis in Swindon and Oxfordshire, UK, and in Milwaukee, Wisconsin, USA, affecting 5000 and > 400,000 people, respectively, brought international attention to the fact that cryptosporidia-contaminated water is the most common source of localised epidemics of the infection [73, 74]. The most common transmission routes for outbreaks include ingesting contaminated drinking or recreational water, consumption of contaminated food (in particular unpasteurised milk), and contact with infected animals (especially during farm visits) [75]. However, outbreaks represent only < 10% of all cases of cryptosporidial infections, while sporadic infections account for the majority of cases of cryptosporidiosis [76–78]. Since outbreaks are more likely to be reported in the literature and media, this section will focus on them in more detail.

**Fig. 6:**
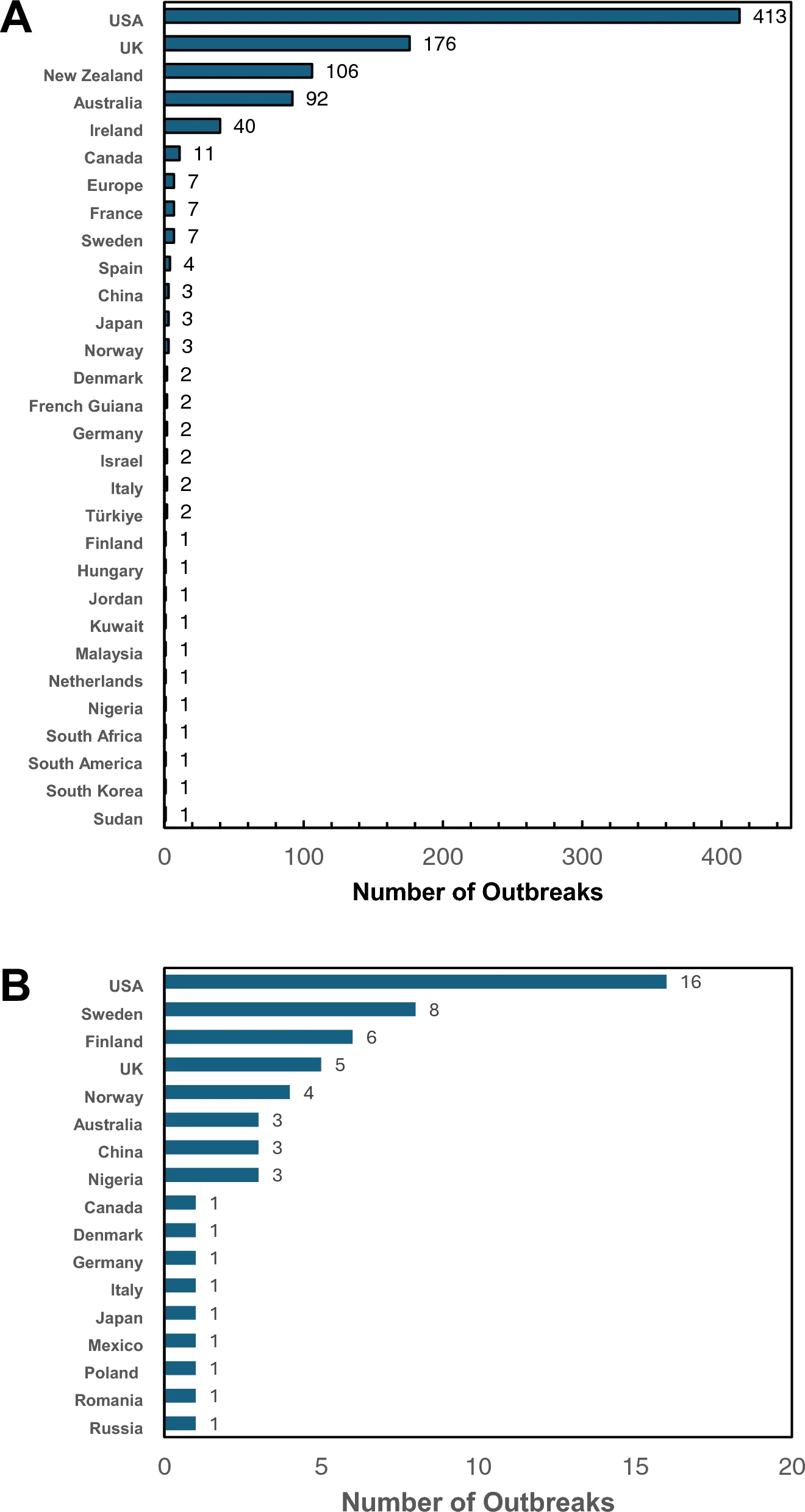
Numbers of waterborne (**A**) and foodborne (**B**) outbreaks of human cryptosporidiosis by country or region. Waterborne outbreaks date are from 1984 to 2022, while foodborne outbreaks date from 1984 to 2024. Data compiled from [74–79, 85]. The figure was created with Excel. The final version of the figure was prepare using PowerPoint

Between 1984 and 2022, at least 895 waterborne outbreaks of human cryptosporidiosis were reported in 27 countries, 1 territory, and 2 regions (Fig. 6a) with a minimum of > 600,000 estimated cases [79–84]. Most of the outbreaks were recorded in the USA (46.1%), followed by the UK (19.1%), New Zealand (11.4%), and Australia (10.3%) (Fig. 6a). The majority of outbreaks were associated with recreational water activities (63.8%), while drinking/tap water was responsible for 13.2% and public water supplies for 9.4% of outbreaks. In 2.4%, the source of the outbreaks was due to other reasons, while in 11.2%, the source was unclear or undefined. In 55.6% of the outbreaks, the *Cryptosporidium* species was not specified, while in 25.2% and 18.0%, the pathogen was identified as *C. parvum* and *C. hominis*, respectively. A few outbreaks (11 or 1.1%) were due to other *Cryptosporidium* species, including *C. cuniculus* [85], *C. ditrichi* [86], *C. tyzzeri* [86], *C. viatorum* [86–88], *C. mortiferum* (*C. chipmunk* genotype I) [86, 89], *C. occultus* [87], *C. baileyi* [88], and *C. felis* [88]. Regarding foodborne human cryptosporidiosis, at least 55 outbreaks in 17 countries (Fig. 6b) with > 9000 estimated cases were reported between 1984 and 2024 [83, 84, 90]. The reason for the lower number of foodborne outbreaks of human cryptosporidiosis is that food is far less likely to be contaminated with oocysts than water and that oocysts rarely survive typical food preservation treatments such as freezing, heating, and drying. Like waterborne cryptosporidiosis, most foodborne outbreaks happened in the USA (29.1%), followed by Sweden (14.5%) and Finland (10.9%). Most foodborne outbreaks were due to contaminated salad (24.5%) and raw/unpasteurised milk (22.4%), while in 18.4% the source was unknown. The remaining 34.7% of outbreaks were caused by contaminated vegetables, sauces, raw/undercooked meat, vegetable/fruit juices, apple cider, and meals at restaurants. In contrast to waterborne cryptosporidiosis, the responsible pathogen in over half of the foodborne outbreaks was *C. parvum* (63.3%), while *C. hominis* played only a minor role (10.0%). In 16.7%, the *Cryptosporidium* species was not identified. Ten percent of the outbreaks were due to other *Cryptosporidium* species, including *C. ubiquitum* [91, 92], *C. ryanae* [92], *C. bovis* [92], *C. xiaoi* [92], and *C. felis* [93].

Noteworthy is the observation that the vast majority of human cryptosporidiosis outbreaks were reported in high-income nations. Only 2.4% (23 out of 950) of all outbreaks occurred in low-income or middle-income countries or regions. This disparity is primarily due to a combination of higher surveillance capabilities, better detection methods, improved public health policies, and specific environmental/recreational factors in industrialised nations compared to low-income and middle-income countries. Furthermore, intensive and large-scale livestock farming, grazing nearby waterbodies, and manure spreading onto fields are additional factors that contribute to a high prevalence of waterborne cryptosporidiosis in developed countries.

### Drug development

**Fig. 7:**
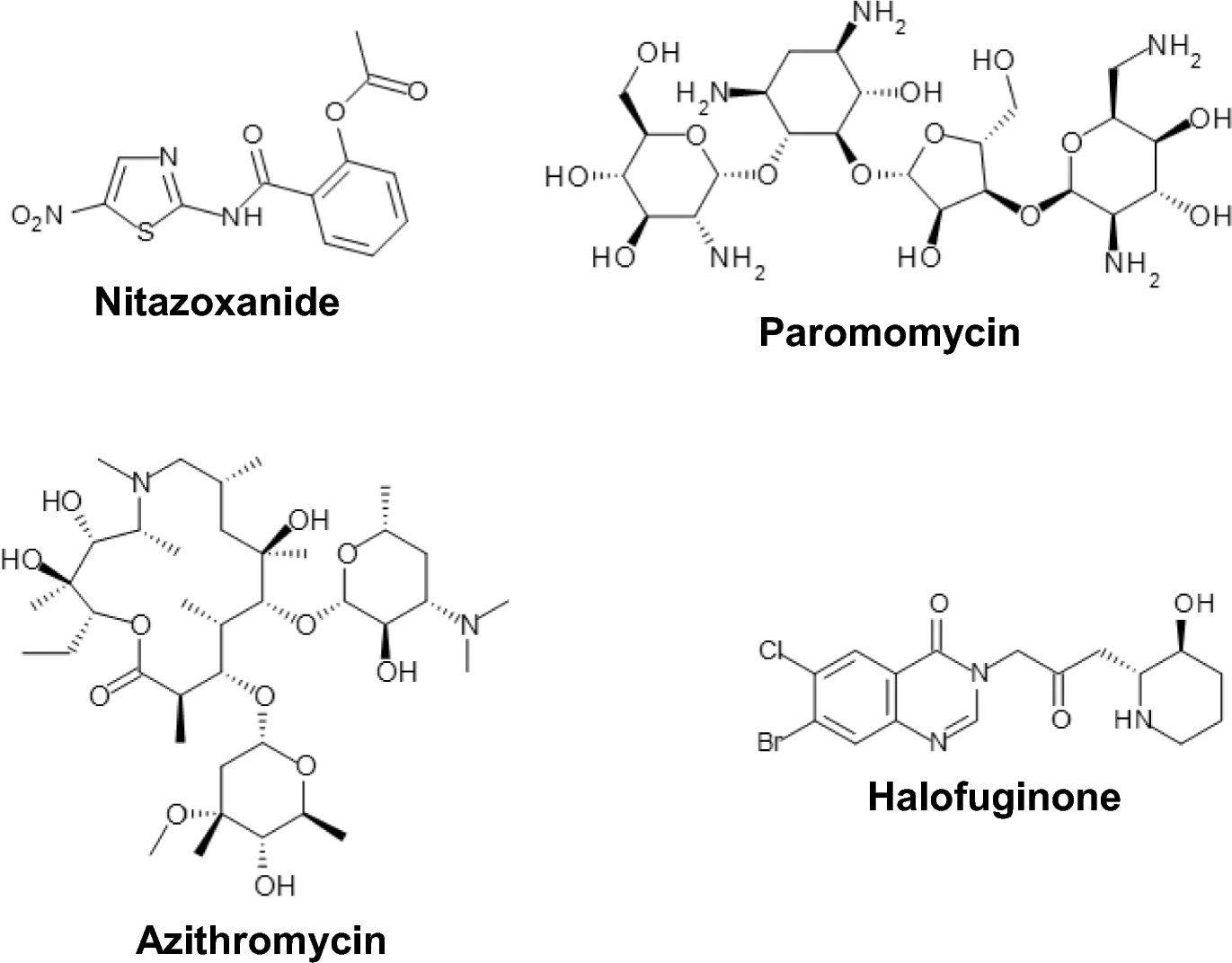
Chemical structures of drugs currently used for the treatment of cryptosporidiosis. The chemical structure were drawn with the software Symyx Draw 3.2. The final version of the figure was prepare using PowerPoint

As cryptosporidiosis is a self-limiting disease in immunocompetent individuals, initial drug development mainly focused on repurposing known anti-parasitic compounds for the treatment of infected patients with a weakened immune system. Since 1990, several anti-parasitic drugs have been tested in clinical trials to determine their efficacy against cryptosporidial infections [94]. However, most of the drugs were found to be not very effective against cryptosporidia when tested in target groups of AIDS patients and young children. So far, nitazoxanide is the only drug that has been approved by the United States Food and Drug Administration (FDA) in 2002 for the treatment of paediatric cryptosporidiosis [95]. Nitazoxanide (Fig. 7) was synthesised in the 1970s initially as a veterinary vermicide on the scaffold of the anthelmintic compound niclosamide by replacing the nitrobenzene ring with a nitrothiazole ring [96]. Eventually, in the late 1990s, it was found that nitazoxanide is effective in cell culture and animal models against *C. parvum* [97, 98], and subsequent clinical trials demonstrated the efficacy of the drug for the treatment of diarrhoea caused by the parasite in adults and children [99, 100]. However, in studies with HIV/AIDS patients, it was found that nitazoxanide is ineffective in reducing the duration or frequency of diarrhoea [101, 102]. The aminoglycoside antibiotic paromomycin (Fig. 7) was shown in the early 1990s to be an effective treatment of cryptosporidial diarrhoea in AIDS patients [103, 104]. Although paromomycin is not licensed by any regulatory authority for the treatment of cryptosporidiosis, it is used as an off-label drug to manage clinical symptoms of cryptosporidial infections in immunocompromised patients and children. Another off-label drug for the treatment of cryptosporidiosis is the macrolide azithromycin (Fig. 7). In the 1990s and early 2000s, this antibiotic was shown to be effective in treating both immunocompetent and immunocompromised children with cryptosporidiosis, with prompt clinical improvement and high rates of parasite clearance [105–107]. However, azithromycin does not seem to be effective in the treatment of cryptosporidiosis in adult AIDS patients. While some studies found that azithromycin had no therapeutic or prophylactic efficacy in the treatment of diarrhoea in AIDS patients infected with cryptosporidia [108, 109], other studies showed that the antibiotic had noticeable clinical and parasitological improvement in individuals with cryptosporidiosis and advanced HIV [110, 111].

For the treatment of cryptosporidiosis in newborn calves, only two drugs, halofuginone lactate and paromomycin, are licensed. Halofuginone (Fig. 7) is a synthetic halogenated derivative of febrifugine, a natural alkaloid found in the Chinese medicinal herb *Dichroa febrifuga* (Chinese quinine or blue evergreen hydrangea), and was approved in 1987 as an anticoccidial for use in poultry [112]. The anti-cryptosporidial activity of halofuginone in dairy calves was shown by the late 1980s and early 1990s [113–115]. Eventually, in 1999, halofuginone was authorised by the European Commission under the name Halocur (halofuginone lactate) for the treatment and prevention of cryptosporidiosis in neonatal calves [116]. The prophylactic efficacy of paromomycin against *C. parvum* infections in dairy calves was shown for the first time in 1993 [117]. However, it took another 16 years before paromomycin was licensed in the European Union (EU) for the treatment of cryptosporidiosis in pre-ruminant calves [118].

### Current situation

Human cryptosporidiosis data for 2025 have only been reported for Australia [119]. After a surge in 2024, cryptosporidiosis cases dropped by 65% to 4870 in 2025, resulting in an average annual incidence of 17.7 per 100,000 population. However, there was a marked geographic variation in the disease, with Tasmania having the lowest case numbers (39) and New South Wales the highest (1325). The next most recent cryptosporidiosis data are from England and New Zealand for the year 2024 [120, 121]. In England, the total number of laboratory-confirmed cryptosporidiosis cases in 2024 was 5708, representing an average annual incidence of 9.9/100000. The lowest and the highest annual incidences of 6.4 and 15.5 per 100,000 were reported for London and the South West, respectively. Although cryptosporidiosis cases were 16.4% lower in 2024 than in 2023, they remained higher than those observed for the years 2015 to 2022. In contrast to England, in New Zealand, the number of cryptosporidiosis cases increased by almost 50% to 1234 in 2024 compared to 2023 notifications, leading to an average incidence of 23.1/100000 annually. For Canada, the latest cryptosporidiosis data are for the year 2023 [122]. Compared with the previous year, an increase of 33% to 1238 cases was recorded for 2023. However, the mean annual incidence was relatively low at 3.3/100000. The most recent cryptosporidiosis data for the USA is from 2022 [123]. A total of 12,609 cases were reported with a mean annual incidence of 3.8/100000. The 2022 reported cases were 40% higher than those published in 2021. The highest incidence rate was recorded in Iowa (14.9/100000), while the lowest was in the District of Columbia (0.4/100000) and Puerto Rico (< 0.1/100000). The latest cryptosporidiosis data from the EU dates back to 2021 [124]. Of the 30 European Economic Area (EEA) states, 19 countries reported 4489 cryptosporidiosis cases in 2021, giving an overall incidence rate of 1.8/100000. The 6 countries, Belgium, Finland, Germany, Ireland, Norway, and Sweden, accounted for 94% of all confirmed cases. The largest number of cases (1502) was recorded in Germany, while the highest incidence rate (16.7/100000) was noted for Ireland. Compared to 2017–2019, cryptosporidiosis cases and incidence rates were considerably lower. For Latin American, African, and Asian countries, there are no statistical data on annual case numbers and incidences of cryptosporidiosis. This is because low-income and middle-income countries lack monitoring and reporting systems for cryptosporidiosis.

The most recent study on the global prevalence of bovine cryptosporidiosis is a meta-analysis published in 2025 [3]. Based on samples from 124,150 cattle, the overall worldwide pooled prevalence of cryptosporidial infections in bovines was 26.5% (95% CI 24.0–26.0%) between 2003 and 2023. The highest prevalence was determined for Europe (33.5%, 95% CI 28.0–39.0%) and the lowest for Africa (21.0%, 95% CI 18.0–24.0). With respect to age groups, the highest global prevalence was noted for pre-weaned calves (37.5%, 95% CI 32.0–43.0%), followed by calves (27.0%, 95% CI 24.0–30.0%) and adult cattle (16.0%, 95% CI 12–20%). Globally, *C. parvum* was the most frequently identified species causing bovine cryptosporidiosis on all continents.

## Conclusions

Cryptosporidia are ancient parasites that evolved with the rise of the vertebrates around 600 million years ago. They are highly specialised Apicomplexans, although they lack the apicoplast characteristic of this group of protozoa. In addition, and in contrast to other Apicomplexans, cryptosporidia have only DNA-lacking mitochondria-related organelles. The loss of these two subcellular organelles is most likely the result of the evolutionary adaptation of cryptosporidia to live in vertebrate hosts.

Palaeoparasitological findings indicate that cryptosporidia have been parasites of humans since ancient times. In this context, however, it is interesting to note that all evidence of cryptosporidia in human specimens comes from the New World. However, the absence of palaeoparasitological evidence of cryptosporidia in human samples in the Old World is not due to researchers not having looked for it. In fact, there are several reports of failed attempts to detect cryptosporidia in human specimens in the Old World (studied and reviewed in [125]). It is also not due to a failure in the detection methods used, as these have been successfully employed to detect cryptosporidia in human specimens in the New World and other gastrointestinal protozoan parasites (*Entamoeba histolytica* and *Giardia duodenalis*; reviewed in [126, 127]) in human samples in the Old World. This asymmetrical record can probably only be explained by taphonomic limitations. For example, while much of the Old World palaeoparasitological records were built by examining samples obtained from latrines, sewers, and burial sediments, the majority of positive cryptosporidia finds in the New World came from desiccated coprolites discovered in dry sites [125], which provided a better taphonomic preservation. Another reason for the lack of Old World palaeoparasitological evidence of cryptosporidia could be that generally a smaller number of samples have been tested in the Eastern Hemisphere [125].

Recent epidemiological data from Canada, the USA, the UK, the EU/EEA, Australia, and New Zealand indicate that cryptosporidiosis cases dropped during the global COVID-19 pandemic in 2020/21 [119–124]. The reduction in reported cases is most likely due to lockdowns and restrictions that impacted the social interaction of people, i.e., less travel, fewer leisure activities, fewer restaurant visits, and fewer farm trips. When the COVID-19 restrictions were lifted, a surge in cryptosporidiosis cases was observed from 2022 to 2024. Since then, cryptosporidiosis cases have been gradually returning to pre-COVID-19 levels. It can be expected that in the next few years, the annual cryptosporidiosis incidence will go back to rates seen before the COVID-19 pandemic.

Although cryptosporidiosis is a self-limiting disease, the few available drugs severely restrict the treatment of the infection. In particular, the only approved drug, nitazoxanide, is ineffective in cryptosporidia-infected immunocompromised patients (e.g., HIV/AIDS patients), who can develop life-threatening diarrhoeal disease. Likewise, the two licensed drugs for bovine cryptosporidiosis are not very effective in the treatment of infected neonatal calves, in which the disease can cause diarrhoea and sometimes even death.

All in all, future efforts should focus on implementing surveillance systems to monitor cryptosporidiosis in low-income and middle-income countries and on the development of effective drugs to treat the disease in both humans and cattle. Effective veterinary drugs against bovine cryptosporidiosis are necessary, as the infection is predominantly transmitted to humans via zoonotic routes.

## Data Availability

Data supporting the main conclusions of this study are included in the manuscript.

## Abbreviations

EEA: European economic area
ELISA: Enzyme-linked immunosorbent assay
EU: European Union
FDA: United States Food and Drug Administration
HSP70: Heat shock protein 70
IFA: Immunofluorescence assay
MRO: Mitochondria-related organelle
